# Supplementary material for: A multiscale approach reveals elaborate circulatory system and intermittent heartbeat in velvet worms (Onychophora)
Source: Commun Biol. 2023 Apr 28;6:468. doi: 10.1038/s42003-023-04797-z (PMC10147947; doi:10.1038/s42003-023-04797-z)
Supplement: Supplementary file 2 — Supplementary Information [file 42003_2023_4797_MOESM2_ESM.pdf]

Supplementary Information

A multiscale approach reveals elaborate circulatory system and intermittent heartbeat in velvet worms (Onychophora)

Henry Jahn, Jörg U. Hammel, Torben Göpel,

Christian S. Wirkner & Georg Mayer

Content:

Supplementary Figures
Supplementary Tables
Supplementary Protocols
Supplementary Code
Supplementary References

Supplementary Figures

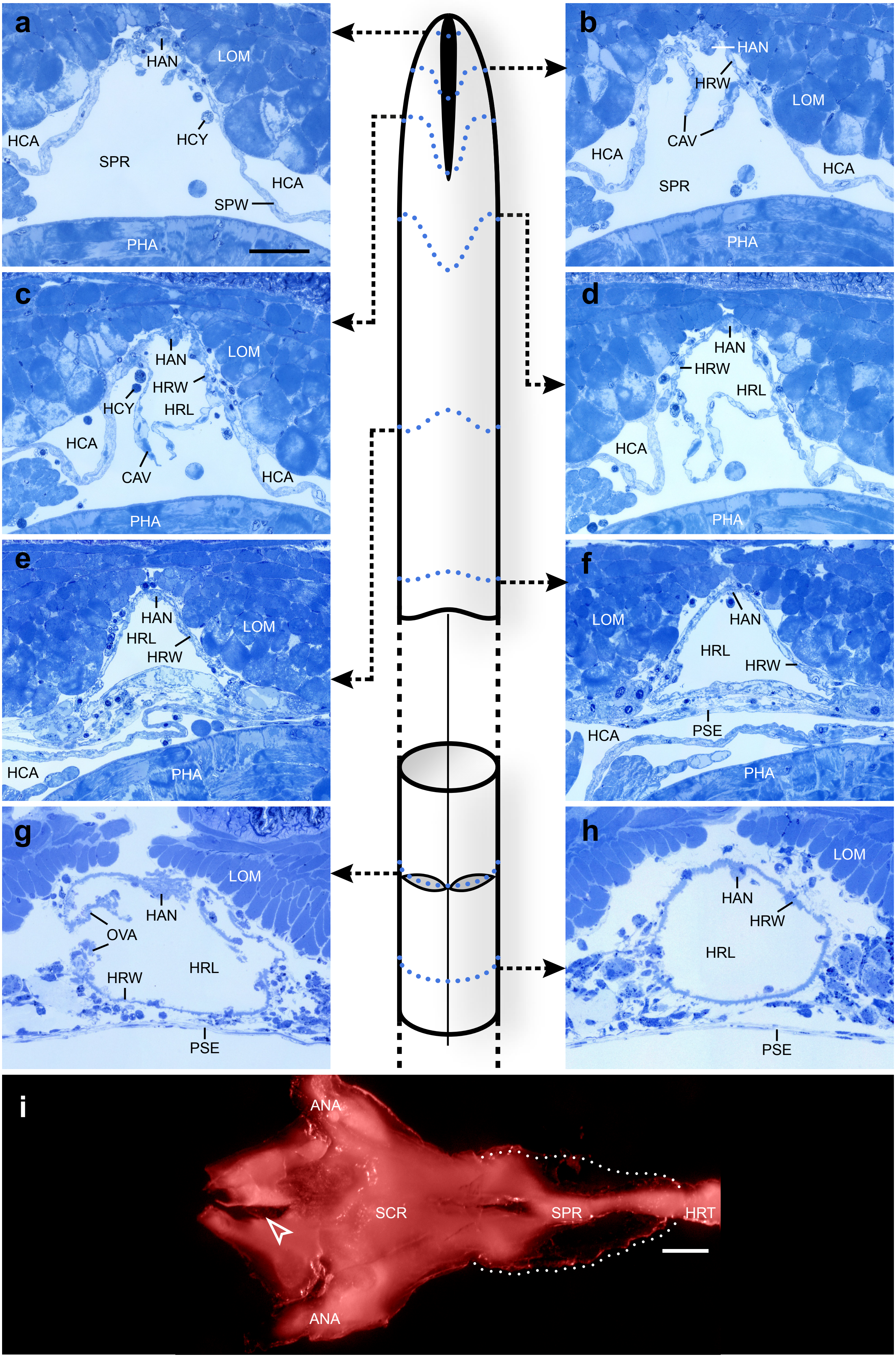

Supplementary Fig. S1: Details of connection area between heart and anterior aorta as well as heart in midbody region of *E. rowelli*. Light micrographs of semi-thin cross section (**a**–**h**) and

corrosion cast (**i**). Dorsal is up in **a**–**h**, anterior is left in **i**. Diagram indicates position of semi-thin sections (blue dotted lines) along the heart (ventral view, anterior is up). Lumen of heart leads into suprapharyngeal region of anterior aorta after passing ventral cardiac valve. Dotted line in **i** demarcates outlines of suprapharyngeal region of anterior aorta. Arrowhead points to indentation of frontal muscle. Abbreviations: ANA, antennal artery; CAV, cardiac valve; HAN, heart nerve; HCA, head cavity; HCY, hemocyte; HRL, lumen of heart; HRW, heart wall, LOM, longitudinal musculature of body wall; OVA, ostial valves; PHA, pharynx; PSE, pericardial septum; SCR, supracerebral region of anterior aorta; SPR, suprapharyngeal region of anterior aorta; SPW, wall of suprapharyngeal region of anterior aorta. Scale bars: 50 µm (**a**–**h**), 300 µm (**i**).

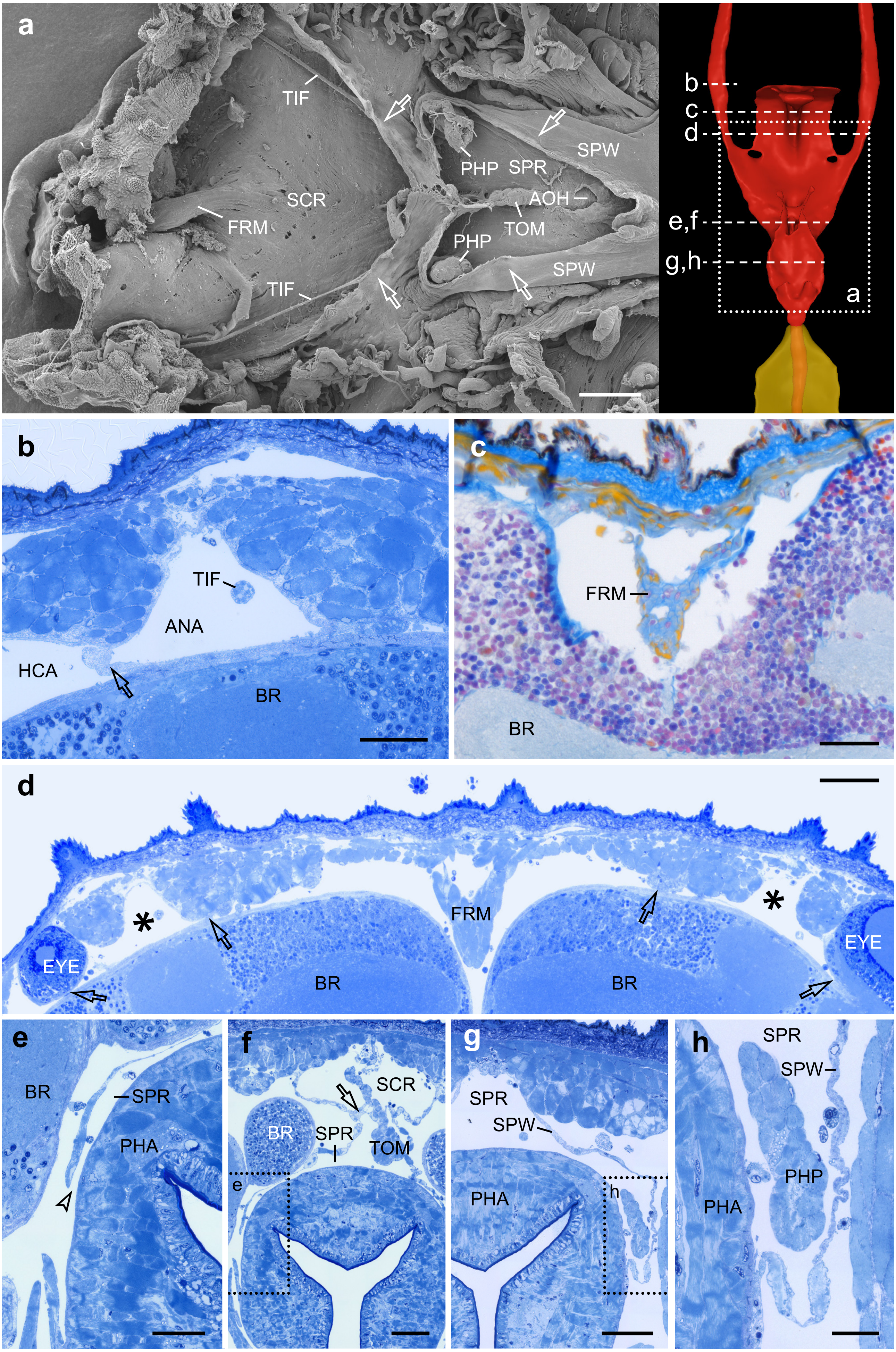

Supplementary Fig. S2: Details of anterior aorta in *E. rowelli*. Scanning electron micrograph (**a**), light micrographs of semi-thin cross sections (**b**,**d**–**h**), and horizontal histological section (**c**).

Dorsal is up in **b**,**d**–**h**; anterior is left in **a** and up in **c**. 3D image in upper right corner illustrates areas depicted in **a**–**h**. **a** Relationship of suprapharyngeal and supracerebral regions of anterior aorta to associated structures. Ventral view; brain and pharynx removed. Note that lumen of anterior aorta is lined by continuous layer of tissue (arrows). **b** Detail of descending antennal artery delimited by strand of connective tissue from head cavity (arrow). **c** Section of frontal muscle, which crosses anterior transition zone of anterior aorta and frontal part of head cavity. **d** Cross section of supracerebral region of anterior aorta delimited by contact zones of dorsal musculature and eyes with dorsal part of brain (arrows). Junctions form anterior aorta to antennal arteries are indicated by asterisks. **e**–**h** Details of suprapharyngeal region of anterior aorta. Dotted lines in **f** and **g** indicate areas shown in **e** and **h**, respectively. **e** Ventral opening of suprapharyngeal region of anterior aorta (arrowhead). **f** Tongue muscle crossing transition zone of suprapharyngeal and supracerebral regions of anterior aorta. Lateral lining of connective tissue fuses with tongue muscle (arrow). **g** Lateral region of suprapharyngeal region of anterior aorta where its lateral lining attaches to pharynx. **h** Pharyngeal protractor crossing ventrolateral part of suprapharyngeal region of anterior aorta. Abbreviations: ANA, antennal artery; AOH, anterior opening of heart; BR, brain; EYE, eye; FRM, frontal muscle; HCA, head cavity; PHA, pharynx; PHP, pharyngeal protractor; SCR, supracerebral region of anterior aorta; SPR, suprapharyngeal region of anterior aorta; SPW, wall of suprapharyngeal region of anterior aorta; TIF, uncharacterized tissue fiber; TOM, tongue muscle. Scale bars: 200 μm (**a**), 40 μm (**b**), 100 μm (**c**,**d**,**f**,**g**), 50 μm (**e**), 30 μm (**h**).

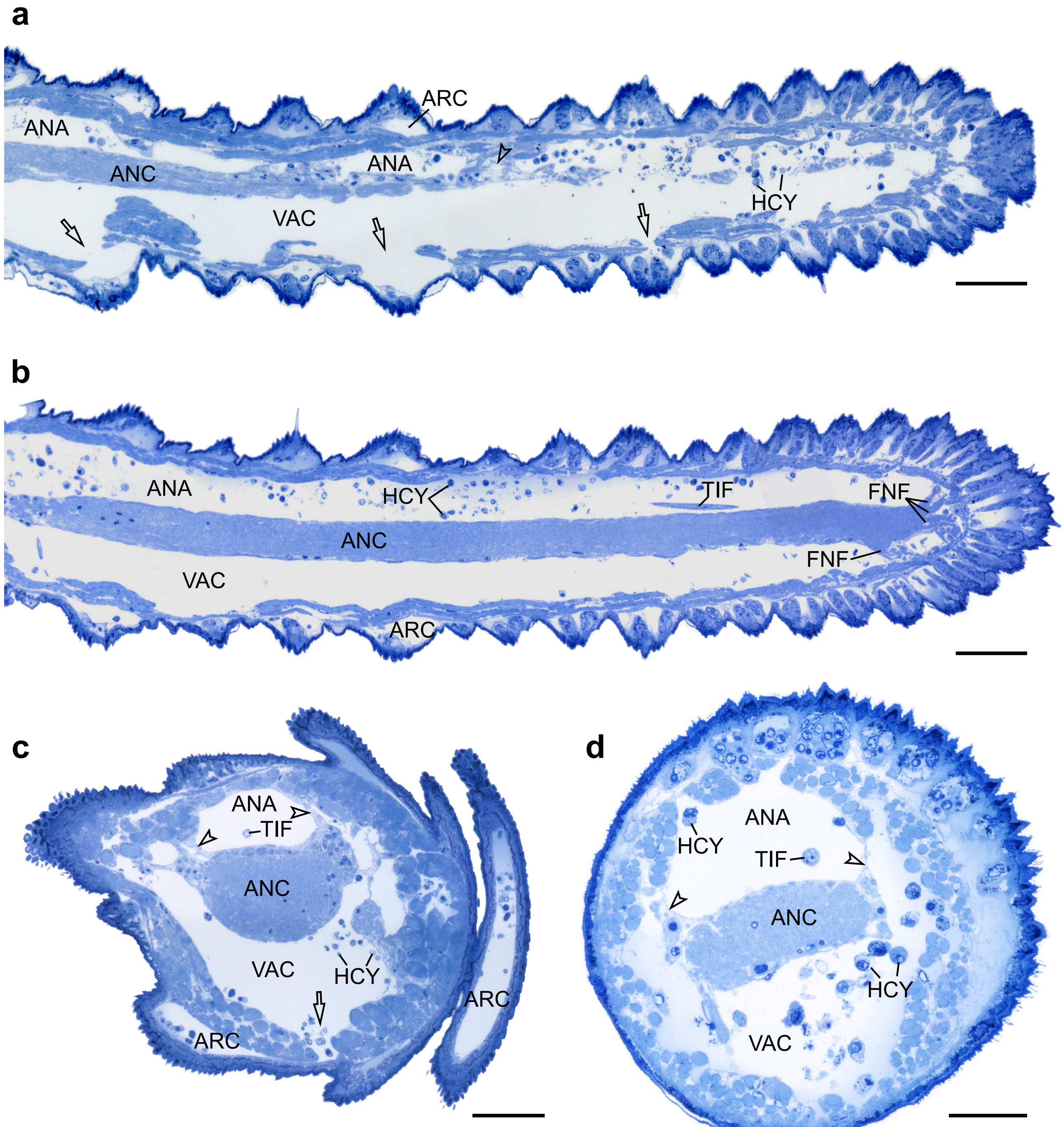

Supplementary Fig. S3: Details of antennal circulatory system in *E. rowelli*. Light micrographs of semi-thin sections. Dorsal is up in all images; proximal is left in **a** and **b**. Arrows point to areas, in which antennal ring channels are confluent with ventral antennal channel. Arrowheads indicate lateral walls of antennal artery that separate it from ventral antennal channel. **a** Sagittal tangential section through lateral region of antenna. **b** Medio-sagittal section of antenna. **c** Cross section through antennal basis. **d** Cross section through middle region of antenna. Note bundles of nerve fibers extending from antennal cord to epidermal cells of antennal tip. Abbreviations: ANA, antennal artery; ANC, antennal nerve cord; ARC, antennal ring channel; FNF, fanning out nerve fibers. HCY, hemocyte; TIF, uncharacterized tissue fiber; VAC, ventral antennal channel; Scale bars: 100 µm (**a**–**d**).

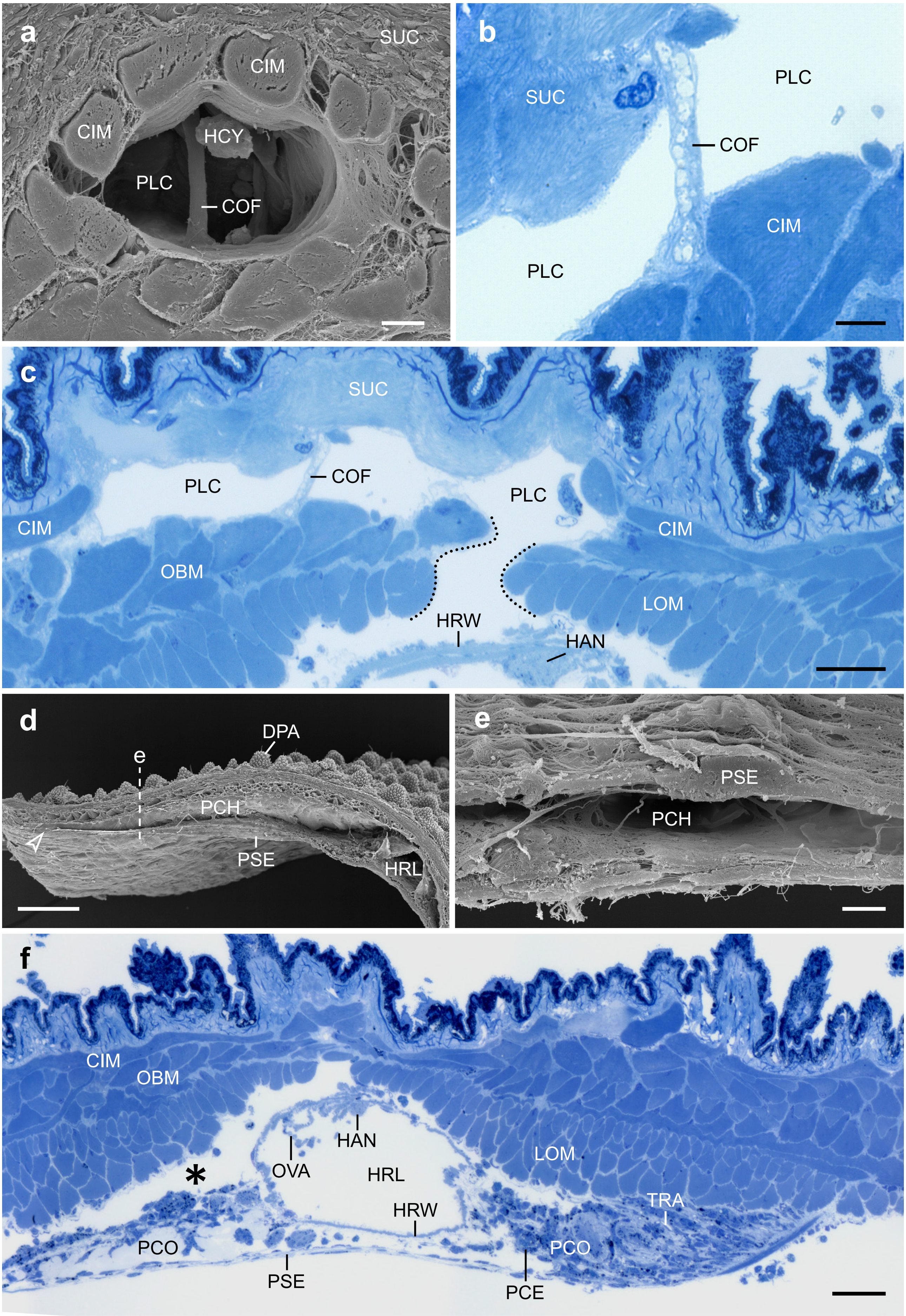

Supplementary Fig. S4: Details of plical and pericardial channels in *E. rowelli*. Scanning electron micrographs (**a**,**d**,**e**) and light micrographs of semi-thin sections (**b**,**c**,**f**). Dorsal is up in **a**–**c**,**e**,**f**; lateral is up in **d**. **a** Cross-sectioned plical channel surrounded by circular musculature and crossed by bundle of collagen fibers. **b** Collagen fibers traversing plical channel. **c** Cross section of

mid-dorsal body region showing plical channel, which is connected via short dorsomedian channel (dotted line) to pericardial sinus. **d** Pericardial channel, which opens into perivisceral cavity (arrowhead). Dashed line indicates approximate region magnified in **e**. **e** Detail of pericardial channel illustrated in **d**. **f** Cross section of mid-dorsal body region demonstrating spatial relationship of heart (with ostial flaps) and channel-like space (asterisk) between pericardial conglomerate of pericardial sinus. Abbreviations: CIM, circular musculature of body wall; COF, collagen fibers; DMC, dorsomedian channel; DPA, dermal papilla; HAN, heart nerve; HCY, hemocyte; HRL, lumen of heart; HRW, heart wall; LOM, longitudinal musculature of body wall; OBM, oblique musculature of body wall; OVA, ostial valve; PCE, pericardial cell, PCH, pericardial channel; PCO, pericardial conglomerate; PLC, plical channel; PSE, pericardial septum; SUC, subepidermal connective tissue; TRA, trachea. Scale bars: 5 μm (**a**,**e**), 10 μm (**b**), 30 μm (**c**), 200 μm (**d**), 50 μm (**f**).

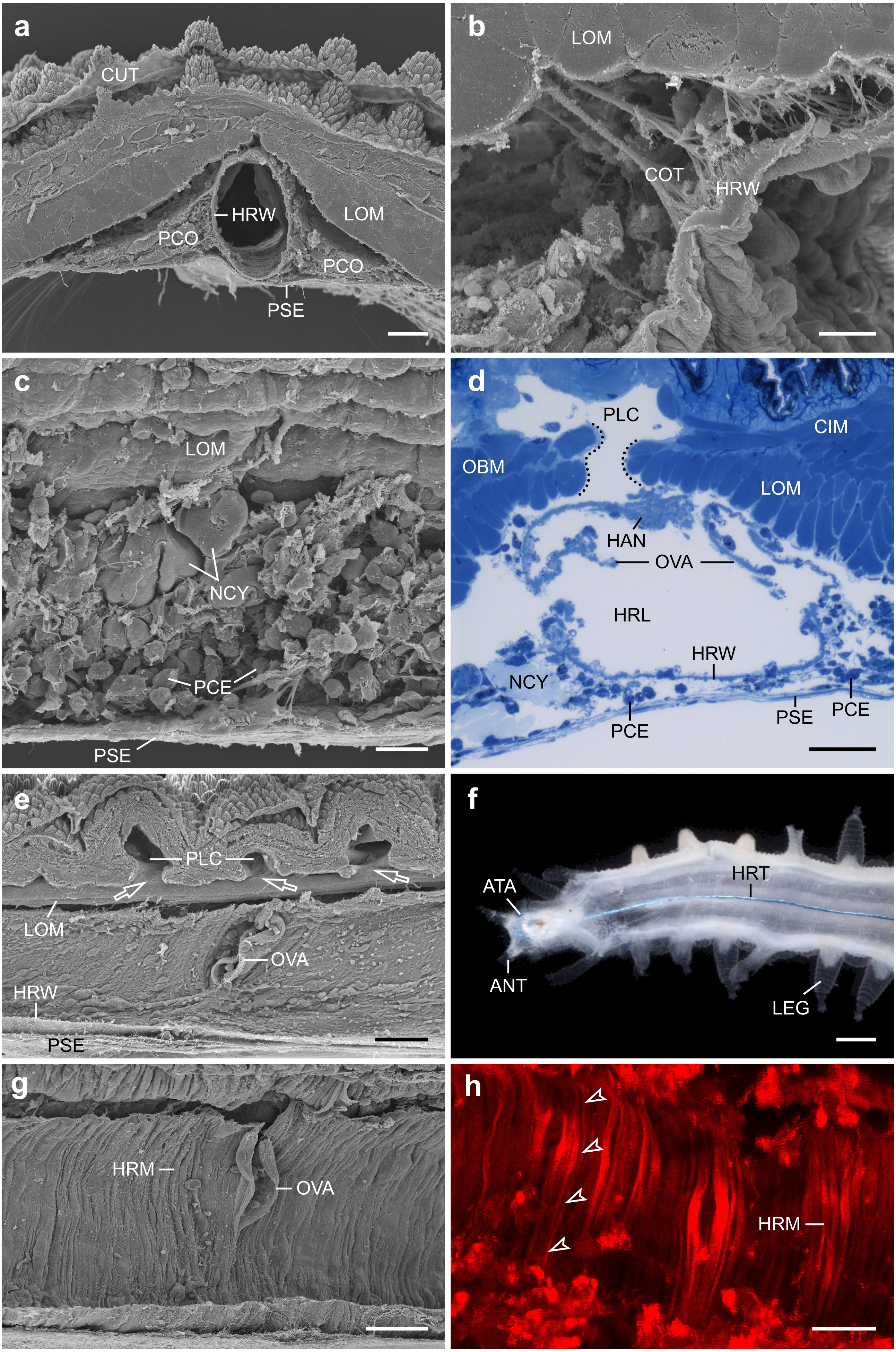

Supplementary Fig. S5: Details of heart and pericardial sinus in *E. rowelli*. Scanning electron micrographs (**a**–**c**,**e**,**g**), light micrograph of semi-thin section (**d**), light micrograph using

stereomicroscope (**f**), and confocal micrograph (**h**). Dorsal is up in **a**–**e**,**g**,**h**; anterior is left in **c**,**e**–**h**. **a** Cross sectioned mid-dorsal body region illustrating position of heart within pericardial sinus, which is ventrally bordered by pericardial septum. **b** Detail of dorsal connective tissue, which attaches heart to longitudinal musculature of body wall. **c** Sagittal section with removed heart showing pericardial conglomerate containing nephrocytes and smaller uncharacterized cells within pericardial sinus. **d** Cross section through heart showing bilateral ostia with flaps projecting into lumen of heart. Dorsomedian channel (black dotted line) links plical channel with pericardial sinus. Note large size and faint staining (methylene blue) of nephrocytes. **e** Medio-sagittal section through heart illustrating ostial valve, dorsomedian channels (arrows) and plical channels. **f** Corrosion cast in partially macerated specimen in ventral view. Note heart and anterior aorta filled with PU4ii casting resin, which appears blue. **g** Internal view of heart wall with ostial flaps. **h** F actin-stained wall of heart illustrating circular arrangement of muscle fibers (arrowheads). Abbreviations: ANT, antenna; ATA, anterior aorta; CIM, circular musculature of body wall; COT, connective tissue attaching heart to dorsolateral longitudinal muscle; CUT, cuticle; HAN, heart nerve; HRL, lumen of heart; HRM, circular musculature of heart; HRT, heart; HRW, heart wall; LEG, leg; LOM, longitudinal musculature of body wall; NCY, nephrocyte; OBM, oblique musculature of body wall; OVA, ostial valves; PCE, pericardial cell, PCO, pericardial conglomerate; PLC, plical channels; PSE, pericardial septum. Scale bars: 50 μm (**a**,**d**,**e**), 10 μm (**b**), 20 μm (**c**), 500 μm (**f**), 40 μm (**g**), 30 μm (**h**).

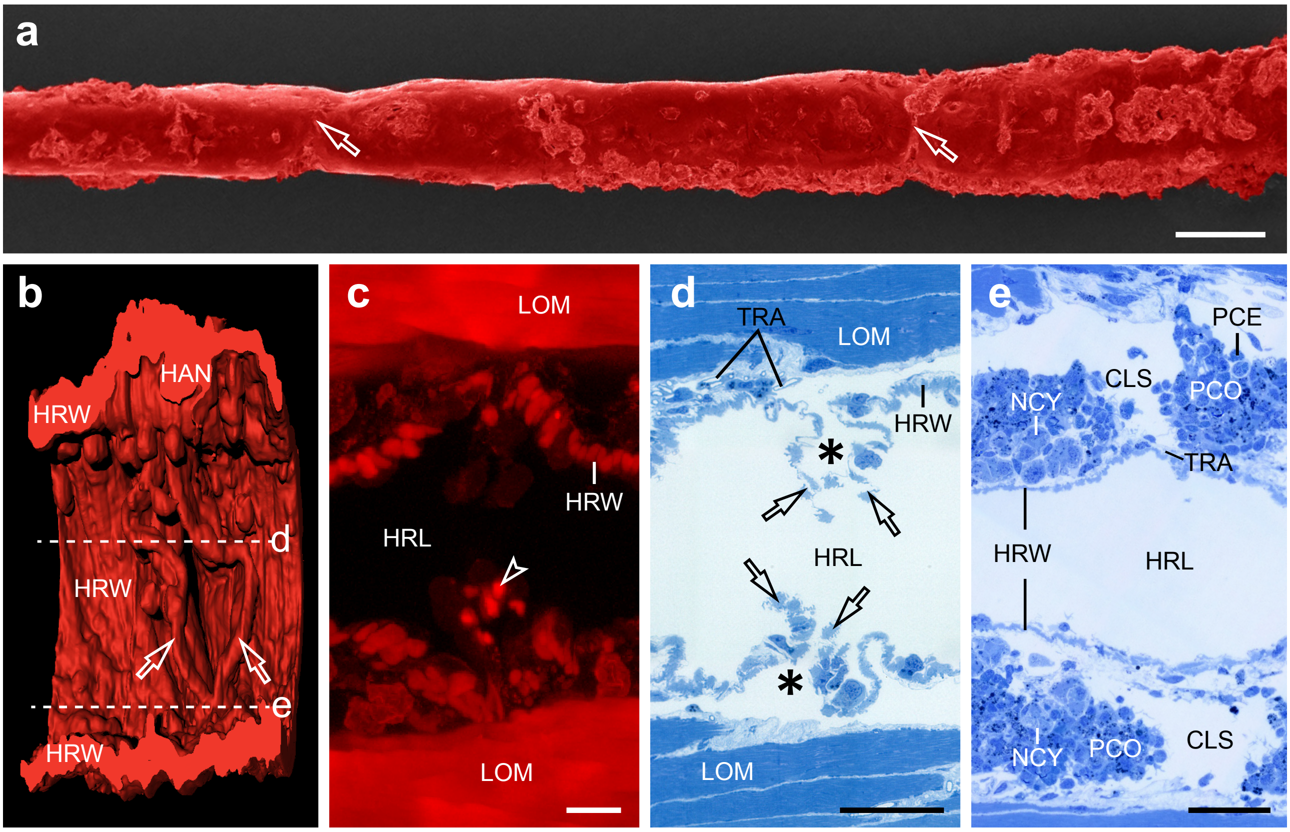

Supplementary Fig. S6: Details of heart and ostia in *E. rowelli*. Pseudo-colored scanning electron micrograph of corrosion cast (**a**), 3D reconstruction based on aligned series of semi-thin sections (**b**), confocal micrograph of horizontal vibratome section (**c**), and light micrographs of horizontal semi-thin sections (**d**,**e**). Anterior is left in **a**–**e**; dorsal is up in **a**,**b**. **a** Corrosion cast of heart lumen with slit-like imprints of ostial valves (arrows). **b** Detail of heart wall with ostium viewed from heart lumen. Dashed lines indicate approximate position of horizontal sections shown in **d** and **e**. **c** Arrangement of muscle fibers in heart wall revealed by F-actin staining. Note prominent signal in valve cells of ostia (arrowhead). **d** Transition area of pericardial sinus (asterisks) into heart lumen between paired ostial valves (arrows). **e** Detail of bilateral segmental channel-like spaces between nephrocytes within pericardial sinus at level of pericardial channels. Note their correspondence in position to ostia. Note also large size and faint staining of nephrocytes with methylene blue. Abbreviations: CLS, channel-like spaces between pericardial conglomerate; HAN, heart nerve; HRL, lumen of heart; HRW, heart wall; LOM, longitudinal musculature of body wall; NCY, nephrocytes; PCE, pericardial cell, PCO, pericardial conglomerate; TRA, tracheae. Scale bars: 200 µm (**a**), 40 µm (**c**), 100 µm (**d**,**e**).

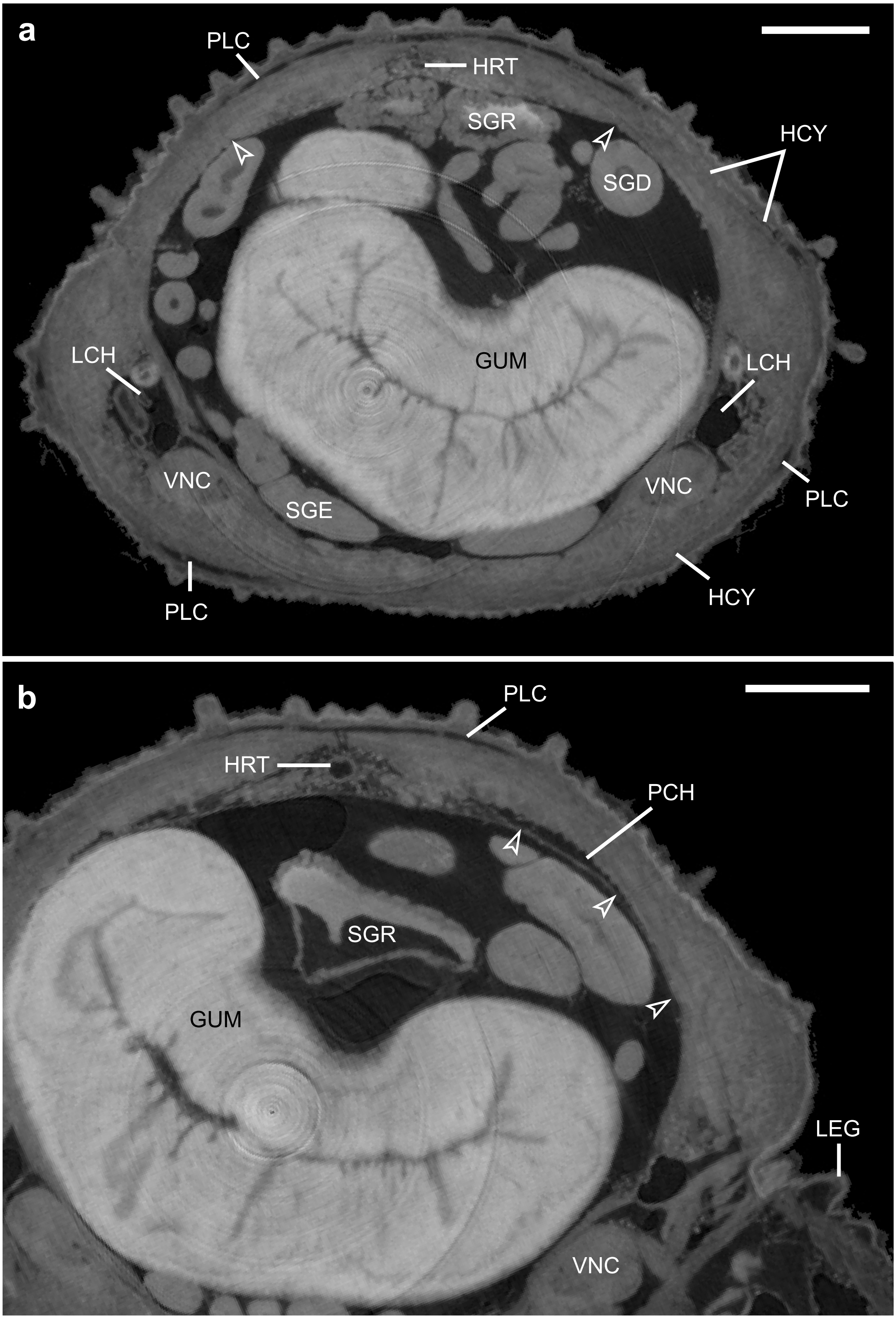

Supplementary Fig. S7: Virtual cross sections of midbody from SR-µCT dataset of *E. rowelli*, which served as basis for 3D reconstructions. Dorsal is up. **a** Cross section of trunk between two leg pairs. Arrowheads indicate attachment points of pericardial septum to musculature of body wall. **b** Cross section of trunk at level of legs. Arrowheads point to extensions of pericardial septum that delimit segmental pericardial channel. Abbreviations: GUM, gut; HCY, hemocyte; HRT, heart; LCH, lateral channel; LEG, leg; PCH, pericardial channel; PLC, plical channel; SGD, secretory duct of slime gland; SGE, end piece of slime gland; SGR, reservoir of slime gland; VNC, ventral nerve cord. Scale bars: 300 µm (**a**,**b**).

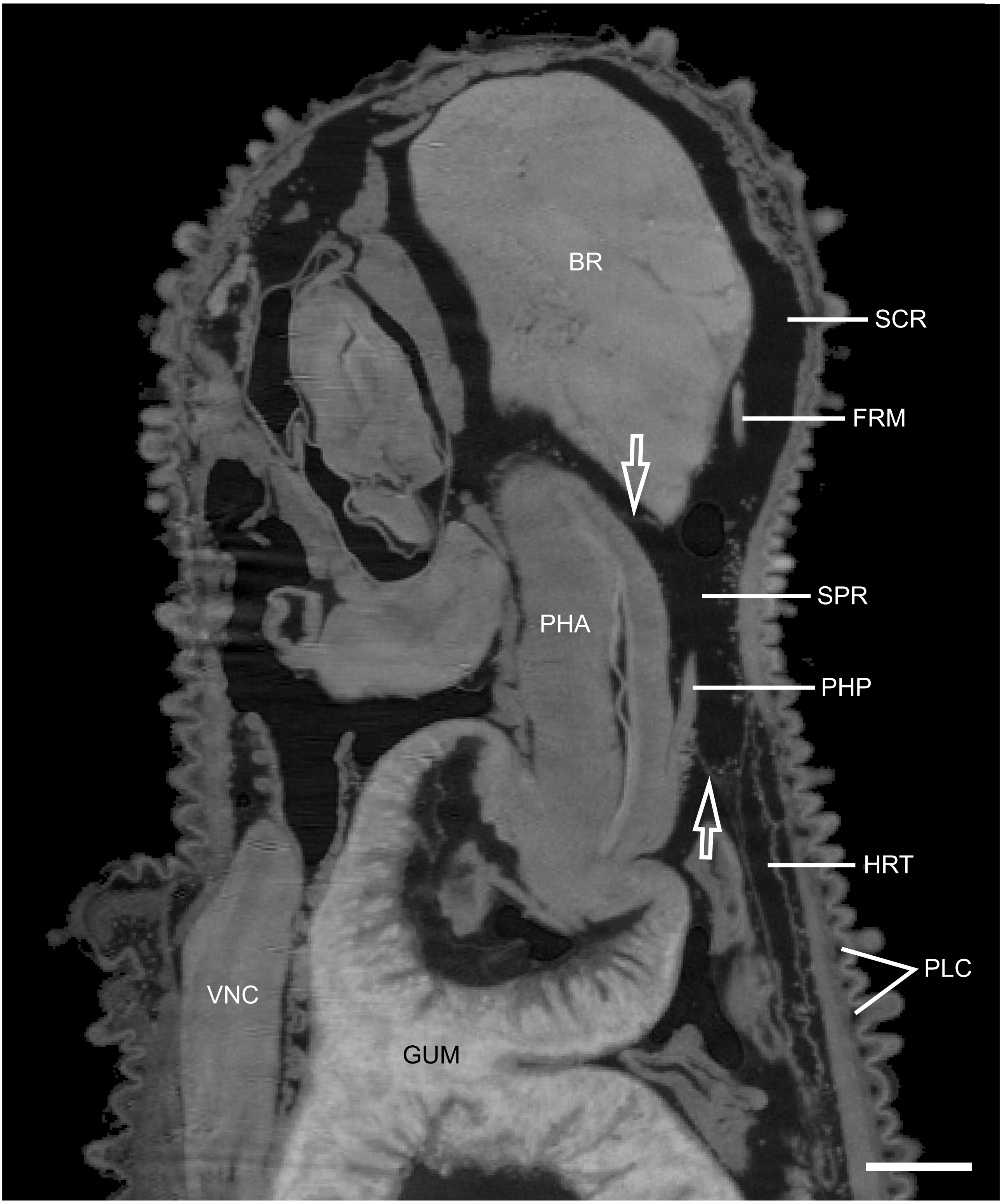

Supplementary Fig. S8: Virtual sagittal section of anterior body region from SR-μCT dataset of *E. rowelli*, which served as basis for 3D reconstructions. Anterior is up, dorsal is right. Nearly mid-sagittal section illustrating spatial relationship of heart and anterior aorta to surrounding structures. Arrows point to linings of anterior aorta. Abbreviations: BR, brain; FRM, frontal muscle; GUM, gut; HRT, heart; PHA, pharynx; PHP, pharyngeal protractor; PLC, plical channel; SCR, supracerebral region of anterior aorta; SPR, suprapharyngeal region of anterior aorta; VNC, ventral nerve cord. Scale bar: 300 μm.

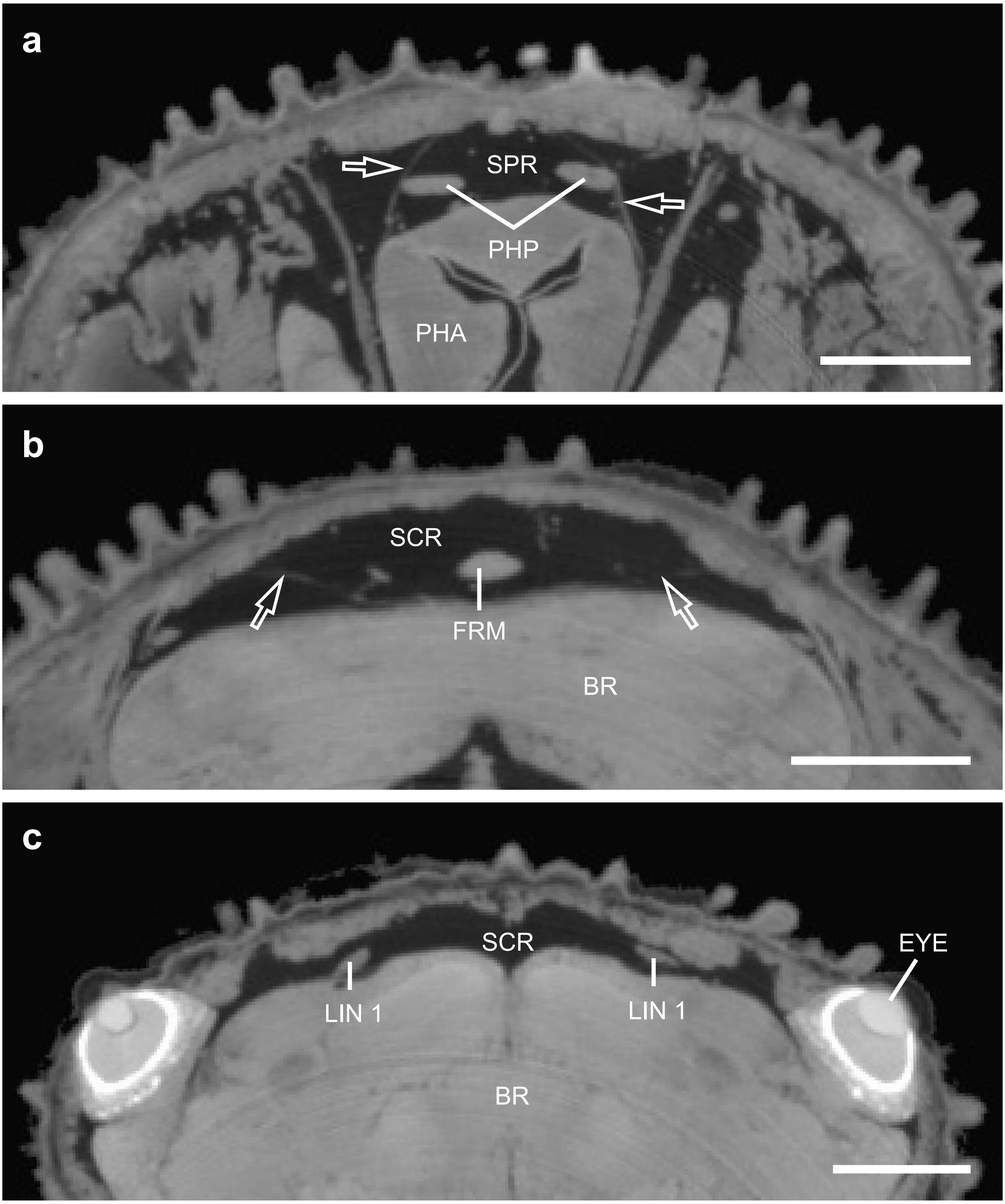

Supplementary Fig. S9: Virtual cross sections of head from SR-µCT dataset of *E. rowelli*, which served as basis for 3D reconstructions. Dorsal is up in **a**–**c**. **a**–**c** Cross sections of suprapharyngeal region of anterior aorta (**a**), and middle (**b**) and anterior parts of supracerebral region of anterior aorta (**c**). Arrows point to linings of vessels. Abbreviations: BR, brain; EYE, eye; FRM, frontal muscle; LIN 1, first pair of oral lip nerves; PHA, pharynx; PHP, pharyngeal protractor; SCR, supracerebral region of anterior aorta; SPR, suprapharyngeal region of anterior aorta. Scale bars: 300 µm (**a**–**c**).

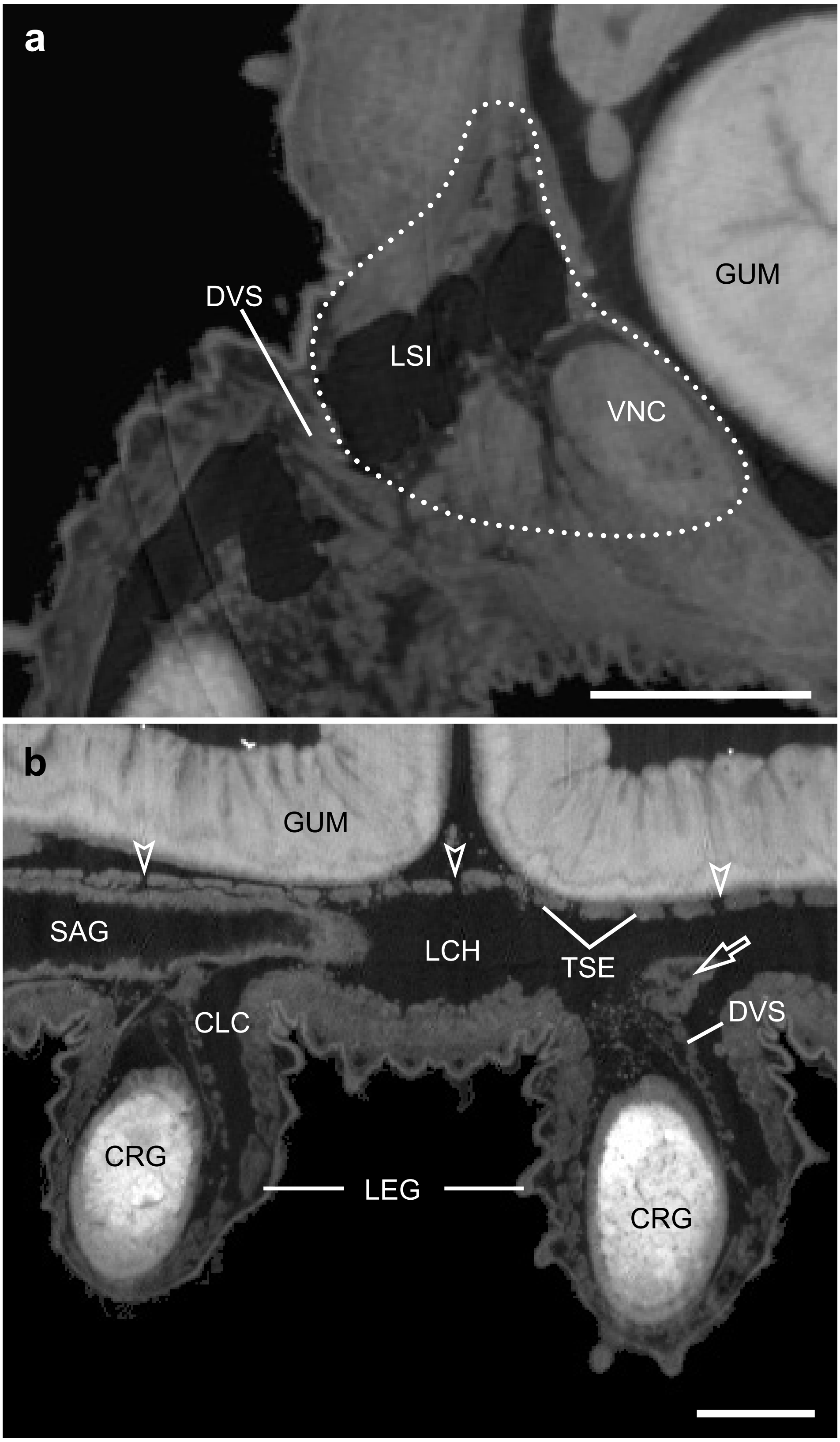

Supplementary Fig. S10: Virtual sections of leg regions from SR-μCT dataset of *E. rowelli*, which served as basis for 3D reconstructions. Dorsal is up in **a**; anterior is left in **b**. **a** Cross section of proximal leg region. Dotted line demarcates lateral sinus harboring ventral nerve cords, nephridia, salivary glands and lateral channels. **b** Horizontal section of two legs showing connections of main leg cavities to lateral channel. Arrow points to leg depressor muscle, which crosses connection between main leg cavity and lateral channel. Note slit-like perforations in transverse septum separating perivisceral sinus from lateral sinuses (arrowheads). Abbreviations: CLC, connection of main leg cavity and lateral channel; CRG, crural gland; DVS, dorsoventral septal muscle of leg; GUM, midgut; LCH, lateral channel; LEG, leg; LSI, lateral sinus; SAG, salivary gland; TSE, transverse septum; VNC, ventral nerve cord. Scale bars: 300 μm (**a**,**b**).

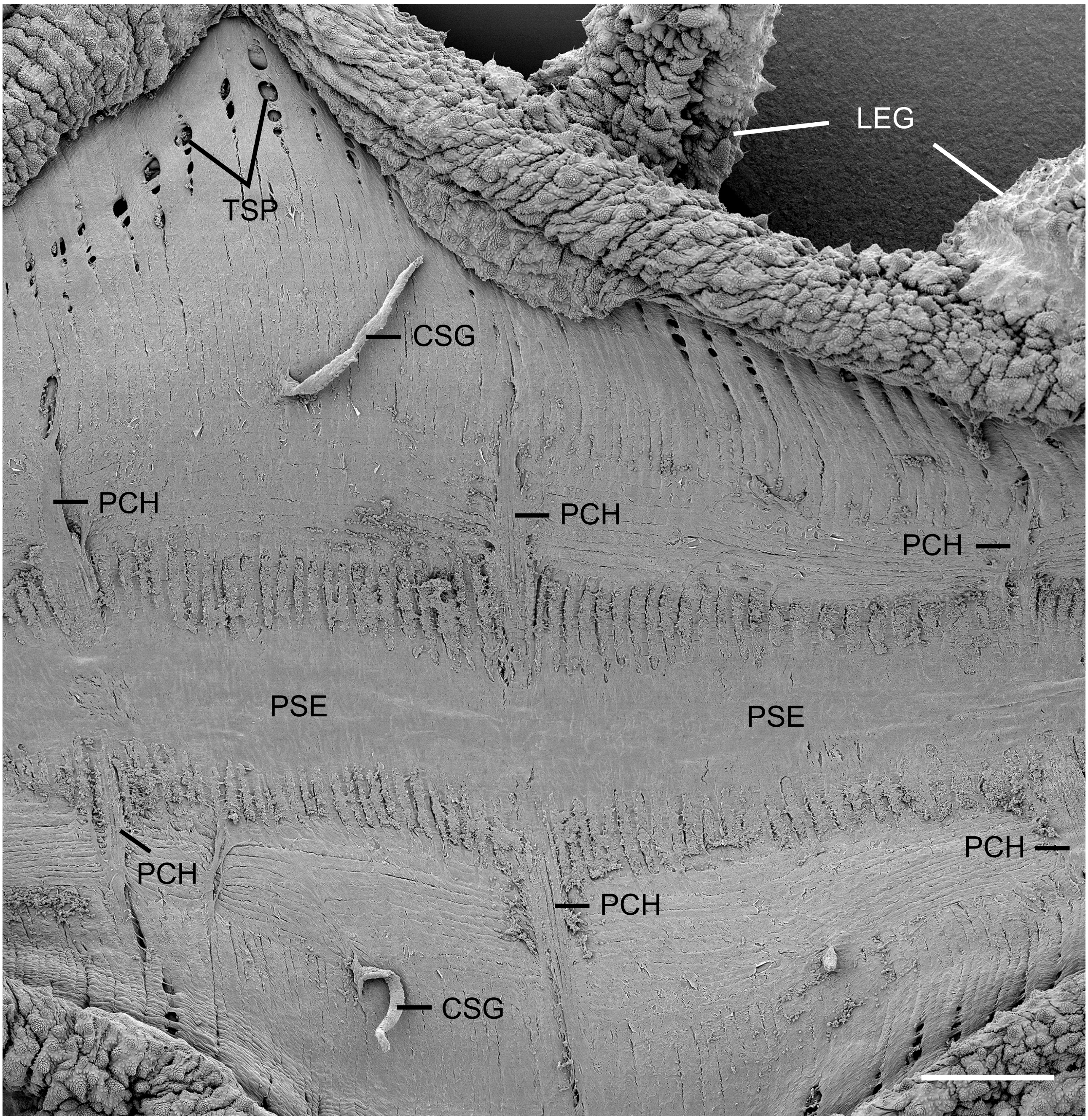

Supplementary Fig. S11: Spatial relationship of pericardial sinus, pericardial septum, pericardial channels, transverse septa, and legs in dissected specimen of *E. rowelli*. Scanning electron micrograph. Ventral view. Anterior is left. Pericardial septum and associated structures. Note segmental arrangement of pericardial channels that correlate in position with legs. Abbreviations: CSG, segregated connecting strand, which links midgut to body wall in intact animal; LEG, leg; PCH, pericardial channel; PSE, pericardial septum; TSP, slit-like perforations in transverse septum. Scale bar: 300 μm.

Supplementary Tables

Supplementary Table 1: Standardized terminology for components of circulatory system and associated tissues and cells in onychophorans and arthropods. Adapted and amended from the Ontology of Arthropod Circulatory Systems database (OArCS, Wirkner et al.[1]). Terms that are highlighted in bold and preceded by an arrow represent references to the corresponding terms in the first column.

Suggested term	Description	Synonym(s)	References for synonyms
Aorta	Part of ➔**vascular system**; longitudinal extension of dorsal ➔**vessel** separated from it by aortic ➔**valve**	–	–
Channel	Narrow, elongated part of ➔**lacunar system**, which directs hemolymph into specific body region; in contrast to ➔**vessels**, channels are not lined with distinct cellular layer	–	–
Circulatory system	Entirety of anatomical structures, cavities and interstitial spaces consisting of ➔**vascular system** and ➔**lacunar system** and filled with ➔**hemolymph**	–	–
Hemocoel	Hemolymph space, which is part of ➔**lacunar system** and contains ➔**hemolymph**	Mixocoel	2, 3
Hemolymph	Body fluid circulating in ➔**circulatory system**, which contains hemolymph plasma (nutrients, hormones, proteins, waste material) and ➔**hemocytes**	Blood	4, 5, 6
Lacuna (plural: lacunae)	Part of ➔**lacunar system**; interstitial space between organs and tissues, which is part of ➔**hemocoel**; in contrast to ➔**sinuses**, lacunae are not segregated by ➔**septa**	–	–
Lacunar system	Part of ➔**circulatory system**, which comprises entirety of ➔**channels**, ➔**lacunae** and ➔**sinuses**	–	–
Septum (plural:septa)	Cell layer or tissue, which segregates a ➔**sinus** from other parts of ➔**lacunar system**	–	–
Sinus	Space of ➔**hemocoel**, which is part of ➔**lacunar system** segregated by a ➔**septum**; sinuses are typically larger than ➔**lacunae**	–	–

Supplementary Table 1: Standardized terminology for components of circulatory system and associated tissues and cells in onychophorans and arthropods. Adapted and amended from the Ontology of Arthropod Circulatory Systems database (OArCS, Wirkner et al.[1]). Terms that are highlighted in bold and preceded by an arrow represent references to the corresponding terms in the first column *(continued)*.

Suggested term	Description	Synonym(s)	References for synonyms
Valve	Anatomical structure, which forms an outlet structure and prevent a reflux of ➔**hemolymph**	–	–
Vascular system	Part of ➔**circulatory system**, which is lined with distinct cell layer; in onychophorans and arthropods, this layer is comprised of non-epithelial cells embedded into extracellular matrix	Blood vascular system	7
		Cardio-vascular system	8
		Hemolymph vascular system	8
Vessel	Anatomical structure, which is part of ➔**vascular system**; in contrast to ➔**lacunae**, ➔**channels** and ➔**sinuses**, vessels are lined with distinct cell layer	Blood vessel	9

Supplementary Table 2: Relative volumes of components of circulatory system of *E. rowelli* based on 3D reconstructions of SR-µCT dataset.

Component	Volume relative to entire circulatory system (%)	Volume relative to entire animal (%)
Circulatory system (total)	100.00	18.82
Vascular system (total)	1.07	0.20
Anterior aorta	0.49	0.09
Heart lumen	0.36	0.07
Antennal arteries	0.22	0.04
Lacunar system (total)	98.93	18.42
Lacunae of perivisceral and lateral sinuses (including leg cavities)	74.30	13.83
Pericardial sinus (including pericardial channels)	14.20	2.65
Plical channels	9.52	1.78
Ventral antennal channels	0.71	0.13
Antennal ring channels	0.20	0.03

Supplementary Protocols

Supplementary Protocol 1: Physiological saline (Robson's saline)

Take for 1 l working solution: 100 ml stock solution A and 1 ml stock solution B and fill up to 1 liter with distilled water. pH 7.3 with NaOH and HCL.
Add 4 g/l glucose to working solution at the day of utilization.

Working solution:

Component	g/l
NaCl	6.26
KCl	0.37
$CaCl_2 * 2 H_2O$	0.48
$MgCl_2 * 6 H_2O$	0.073
$NaHCO_3$	0.005
$NaH_2PO_4 * 2 H_2O$	0.00055

Stock solution A:

Component	g/l
NaCl	62.6
KCl	3.7
$CaCl_2 * 2 H_2O$	4.8
$MgCl_2 * 6 H_2O$	0.73

Stock solution B:

Component	g/l
$NaHCO_3$	5.0
$NaH_2PO_4 * 2 H_2O$	0.55

Supplementary Protocol 2: Phosphate-buffered saline (PBS, 10x)

1.37 M NaCl
27 mM KCl
14 mM KH_2PO_4
43 mM Na_2HPO_4
pH 7.4 with NaOH

Dilute this solution 1:10 before usage.

Supplementary Protocol 3: Fixative Bouin's fluid for histology (modified after Dubosq-Brasil)

Stock solution: 10 g picric acid in 1 l of 70 % ethanol
Working solution: 80 ml stock solution + 20 ml of 35 % methanol + 2 ml acetic acid

Supplementary Code

Supplementary Code 1: Macro to count pixels on binary image stacks using Fiji[10]

```
searchValue = 255;
countValue = 0;
for(i = 1; i <= nSlices; i++)
{setSlice(i);
for(x = 0; x < getWidth(); x++)
{for(y = 0; y < getHeight(); y++)
{if(getPixel(x,y) == searchValue)
{countValue += 1;}}}
print(countValue);
countValue = 0;}
```

Supplementary References

1. Wirkner, C. S. et al. The first organ-based ontology for arthropods (Ontology of Arthropod Circulatory Systems—OArCS) and its integration into a novel formalization scheme for morphological descriptions. *Syst. Biol.* **66**, 754–768 (2017).

2. Bartolomaeus, T. & Ruhberg, H. Ultrastructure of the body cavity lining in embryos of *Epiperipatus biolleyi* (Onychophora, Peripatidae)—a comparison with annelid larvae. *Invertebr. Biol.* **118**, 165–174 (1999).

3. Mayer, G., Ruhberg, H. & Bartolomaeus, T. When an epithelium ceases to exist—an ultrastructural study on the fate of the embryonic coelom in *Epiperipatus biolleyi* (Onychophora, Peripatidae). *Acta Zool.* **85**, 163–170 (2004).

4. Robson, E. A., Lockwood, A. P. M. & Ralph, R. Composition of the blood in Onychophora. *Nature* **209**, 533 (1966).

5. Lane, N. J. & Campiglia, S. S. The lack of a structured blood-brain barrier in the onychophoran *Peripatus acacioi*. *J. Neurocytol.* **16**, 93–104 (1987).

6. Campiglia, S. & Lavallard, R. Pression hydrostatique de l'hémolymphe chez *Peripatus acacioi* Marcus et Marcus (Onychophore). *B. Fisiol. Anim.* **2**, 9–22 (1978).

7. Monahan-Earley, R., Dvorak, A. M. & Aird, W. C. Evolutionary origins of the blood vascular system and endothelium. *J. Thromb. Haemost.* **11**, 46–66 (2013).

8. Wirkner, C. S., Tögel, M. & Pass, G. The arthropod circulatory system. in *Arthropod Biology and Evolution: Molecules, Development, Morphology* (eds. A. Minelli, G. Boxshall, & G. Fusco) 343–391 (Springer, 2013).

9. Federighi, H. The blood-vessels of annelids. *Proc. Natl. Acad. Sci. U.S.A.* **13**, 639–641 (1927).

10. Schindelin, J. et al. Fiji: an open-source platform for biological-image analysis. *Nat. Meth.* **9**, 676–682 (2012).
