# Supplementary material for: A multiscale approach reveals elaborate circulatory system and intermittent heartbeat in velvet worms (Onychophora)
Source: Commun Biol. 2023 Apr 28;6:468. doi: 10.1038/s42003-023-04797-z (PMC10147947; doi:10.1038/s42003-023-04797-z)
Supplement: Supplementary file 3 — Description of Additional Supplementary Files [file 42003_2023_4797_MOESM3_ESM.docx]

**Description of Additional Supplementary Files**

File name: Supplementary Data 1

Description: Posterior end of heart in *E. rowelli*: Image stack of series of sagittal histological sections stained with Azan. Dorsal is up; posterior is right.

File name: Supplementary Data 2

Description: Measurements of heart contraction pattern in *E. rowelli*: Source data for graphs of Figure 8 b–d.

File name: Supplementary Movie 1

Description: Circulatory system of *E. rowelli*: Overview of vascular and lacunar parts of 3D- reconstructed circulatory system.

File name: Supplementary Movie 2

Description: Heart contraction in *E. rowelli*: In vivo recording of contracting heart (top) with plotted diameter measurements (bottom).

File name: Supplementary Movie 3

Description: Movement of ostia in *E. rowelli*: In vivo recording of heart contraction showing open/close mechanism of ostial valves. Note hemolymph flow as evidenced by floating hemocytes.

File name: Supplementary Movie 4

Description: Main body cavity of *E. rowelli*: Fly-through movie of 3D reconstruction showing overview of main body cavity and associated main leg cavities and lacunae.

File name: Supplementary Movie 5

Description: Details of anterior aorta in *E. rowelli*: 3D representation of anterior aorta and its position within the head.

File name: Supplementary Movie 6

Description: Lacunar system in trunk of *E. rowelli*: 3D representation of plical channels, pericardial channels, heart and pericardial sinus. Main body cavity is not shown.

File name: Supplementary Movie 7

Description: Autonomous heart contractions in *E. rowelli*: Dissected dorsal body wall with heart in physiological saline at different magnifications. Note ongoing contractions of heart despite missing connection to brain and ventral nerve cords.
