# Supplementary material for: A multiscale approach reveals elaborate circulatory system and intermittent heartbeat in velvet worms (Onychophora)
Source: Commun Biol. 2023 Apr 28;6:468. doi: 10.1038/s42003-023-04797-z (PMC10147947; doi:10.1038/s42003-023-04797-z)
Supplement: Supplementary file 13 — Reporting Summary [file 42003_2023_4797_MOESM13_ESM.pdf]

nature portfolio

Corresponding author(s): Henry Jahn, Georg Mayer

Last updated by author(s): Mar 13, 2023

Reporting Summary

Nature Portfolio wishes to improve the reproducibility of the work that we publish. This form provides structure for consistency and transparency in reporting. For further information on Nature Portfolio policies, see our Editorial Policies and the Editorial Policy Checklist.

Statistics

For all statistical analyses, confirm that the following items are present in the figure legend, table legend, main text, or Methods section.

n/a	Confirmed	
☒	☐	The exact sample size (*n*) for each experimental group/condition, given as a discrete number and unit of measurement
☒	☐	A statement on whether measurements were taken from distinct samples or whether the same sample was measured repeatedly
☒	☐	The statistical test(s) used AND whether they are one- or two-sided<br>*Only common tests should be described solely by name; describe more complex techniques in the Methods section.*
☒	☐	A description of all covariates tested
☒	☐	A description of any assumptions or corrections, such as tests of normality and adjustment for multiple comparisons
☒	☐	A full description of the statistical parameters including central tendency (e.g. means) or other basic estimates (e.g. regression coefficient) AND variation (e.g. standard deviation) or associated estimates of uncertainty (e.g. confidence intervals)
☒	☐	For null hypothesis testing, the test statistic (e.g. *F*, *t*, *r*) with confidence intervals, effect sizes, degrees of freedom and *P* value noted<br>*Give P values as exact values whenever suitable.*
☒	☐	For Bayesian analysis, information on the choice of priors and Markov chain Monte Carlo settings
☒	☐	For hierarchical and complex designs, identification of the appropriate level for tests and full reporting of outcomes
☒	☐	Estimates of effect sizes (e.g. Cohen's *d*, Pearson's *r*), indicating how they were calculated

Our web collection on statistics for biologists contains articles on many of the points above.

Software and code

Policy information about availability of computer code

Data collection	Heart contraction videos were recorded with the stereomicroscope (Axio Zoom V16) equipped with a digital camera (Axiocam 503 color digital camera) and exported via the software ZEN 2012 blue edition version 1.1.2.0 (Carl Zeiss Microimaging GmbH).
Data analysis	The code to run the FIJI-macro used for counting white pixels of binary image stack extracted from heart contraction videos is available in the Supplementary Information.

For manuscripts utilizing custom algorithms or software that are central to the research but not yet described in published literature, software must be made available to editors and reviewers. We strongly encourage code deposition in a community repository (e.g. GitHub). See the Nature Portfolio guidelines for submitting code & software for further information.

Data

Policy information about availability of data

All manuscripts must include a data availability statement. This statement should provide the following information, where applicable:

- Accession codes, unique identifiers, or web links for publicly available datasets
- A description of any restrictions on data availability
- For clinical datasets or third party data, please ensure that the statement adheres to our policy

Data of this study are presented in the article and the Supplementary Information (Supplementary Code, Supplementary Data, Supplementary Figures, Supplementary Protocols and Supplementary Table). Source data of Fig. 9 b–d are available in Supplementary Data 2. The image stack of the posteriorly blind

ending heart and seven additional movies are available in the figshare-repository (DOI: 10.6084/m9.figshare.22233493). Further supporting data are available from the corresponding author upon reasonable request.

Human research participants

Policy information about studies involving human research participants and Sex and Gender in Research.

Reporting on sex and gender	*Use the terms sex (biological attribute) and gender (shaped by social and cultural circumstances) carefully in order to avoid confusing both terms. Indicate if findings apply to only one sex or gender; describe whether sex and gender were considered in study design whether sex and/or gender was determined based on self-reporting or assigned and methods used. Provide in the source data disaggregated sex and gender data where this information has been collected, and consent has been obtained for sharing of individual-level data; provide overall numbers in this Reporting Summary. Please state if this information has not been collected. Report sex- and gender-based analyses where performed, justify reasons for lack of sex- and gender-based analysis.*
Population characteristics	*Describe the covariate-relevant population characteristics of the human research participants (e.g. age, genotypic information, past and current diagnosis and treatment categories). If you filled out the behavioural & social sciences study design questions and have nothing to add here, write "See above."*
Recruitment	*Describe how participants were recruited. Outline any potential self-selection bias or other biases that may be present and how these are likely to impact results.*
Ethics oversight	*Identify the organization(s) that approved the study protocol.*

Note that full information on the approval of the study protocol must also be provided in the manuscript.

Field-specific reporting

Please select the one below that is the best fit for your research. If you are not sure, read the appropriate sections before making your selection.

☐ Life sciences ☐ Behavioural & social sciences ☒ Ecological, evolutionary & environmental sciences

For a reference copy of the document with all sections, see nature.com/documents/nr-reporting-summary-flat.pdf

Ecological, evolutionary & environmental sciences study design

All studies must disclose on these points even when the disclosure is negative.

Study description	The study was designed to investigate all parts of the circulatory system of onychophorans. Morphological data were obtained by SR-µCT imaging, 3D reconstruction, light microscopy, scannning electron microscopy and confocal laser scanning microscopy. Functional parameters of the heart beat were measured on the basis of video recordings. The number of experimental units were kept low as the animal quantity is very limited.
Research sample	We choose Euperipatoides rowelli (Peripatopsidae) for our studies on onychophorans. This species is the model organism of our laboratory and it is easy to cultivate under constant conditions in a climate cabinet.
Sampling strategy	We choose the sample size as low as possible because the specimen quantity is very limited. Each experiment needs new specimens. Different methods provided the same results as described in the manuscript.
Data collection	SR-µCT data were generated at DESY Beamline P05 (Hamburg), microscopic data were generated as described in the manuscript.
Timing and spatial scale	SR-µCT data were generated in 2015. Three-dimensional reconstruction, microscopic imaging and data analyses were accomplished in the years 2015 to 2022.
Data exclusions	No data were excluded from the analyses.
Reproducibility	We did not measure the reproducibility. Corrosion cast experiments needed 5 trials to get experiences with the handling. Three-dimensional reconstruction, video recordings and microscopic imaging were successful and provided for all attempts to repeat the experiments the same results.
Randomization	A randomization was not relevant for our study as we expect that morphological characters regarding the circulatory system are identical/very similar across individuals of the species investigated.
Blinding	Blinding was not possible in this study as we choose the sample size as low as possible because the specimen quantity of velvet worms is very limited. Each experiment needs new specimens. Different morphological methods provided the same results as described in the manuscript.

Did the study involve field work? ☐ Yes ☒ No

Reporting for specific materials, systems and methods

We require information from authors about some types of materials, experimental systems and methods used in many studies. Here, indicate whether each material, system or method listed is relevant to your study. If you are not sure if a list item applies to your research, read the appropriate section before selecting a response.

Materials & experimental systems

n/a	Involved in the study
☒	☐ Antibodies
☒	☐ Eukaryotic cell lines
☒	☐ Palaeontology and archaeology
☐	☒ Animals and other organisms
☒	☐ Clinical data
☒	☐ Dual use research of concern

Methods

n/a	Involved in the study
☒	☐ ChIP-seq
☒	☐ Flow cytometry
☒	☐ MRI-based neuroimaging

Animals and other research organisms

Policy information about studies involving animals; ARRIVE guidelines recommended for reporting animal research, and Sex and Gender in Research

Laboratory animals	Euperipatoides rowelli (Peripatopsidae, Onychophora), age: few months to 4 years
Wild animals	The study did not involve wild animals. All used specimens were born in the laboratory.
Reporting on sex	The determination of the sex was irrelevant for the analyses relating to the circulatory system.
Field-collected samples	Lab animals were kept in a climate cabinet under constant conditions (temperature 18°C and humidity 80%) and at a photoperiod simulating day and night rhythm.
Ethics oversight	No ethical approval or guidance was required.

Note that full information on the approval of the study protocol must also be provided in the manuscript.
